# Rosai-Dorfman Disease of the Spine: A Case Report of a Rare Disease and Review of the Literature

**DOI:** 10.7759/cureus.26317

**Published:** 2022-06-25

**Authors:** Rabia Bahauddin, Alaa Al-Taie, Fatima Al-Khafaji, Ali Barah

**Affiliations:** 1 Radiology, Hamad General Hospital, Doha, QAT; 2 Clinical Radiology, Hamad General Hospital, Doha, QAT

**Keywords:** rosai-dorfman disease, sinus histiocytosis with massive lymphadenopathy, destombes–rosai–dorfman disease, magnetic resonance imaging, computed tomography, spine

## Abstract

Rosai-Dorfman disease (also known as sinus histiocytosis with massive lymphadenopathy or Rosai-Dorfman-Destombes disease) is a rare reactive histiocytic disease, classically involving the lymph nodes of the neck, but it can also occur in extranodal sites. Isolated spinal involvement is rare but important to identify as it can mimic malignancy with males being affected more than females. We present a case of a 52-year-old female patient who had breast cancer and was admitted with long-standing anal and sacral pain. The MRI lumbar spine showed a mass concerning for metastasis. CT-guided biopsy results showed sheets of histiocyte-like cells, some of which were positive with S100 and showed emperipolesis along with plasma cells, lymphocytes, and neutrophils. Features were in keeping with Rosai-Dorfman syndrome. The patient responded to steroids. The familiarity with this entity saved the patient from going through the agony of this being considered a metastasis of her primary malignancy.

## Introduction

Rosai-Dorfman disease (RDD) was first described in 1969 by Rosai and Dorfman, the etiology of which is mostly unknown [1]. It is also known as sinus histiocytosis with massive lymphadenopathy (SHML). Involvement of the central nervous system without involving other extranodal sites is very rare. Even rarer is isolated spinal involvement, occurring in 20-25% of RDD patients with CNS disease [2]. Diagnosis is a combination of histopathological and radiological findings. Our case report is of a secluded spinal Rosai-Dorfman disease. 

## Case presentation

**Figure 1:**
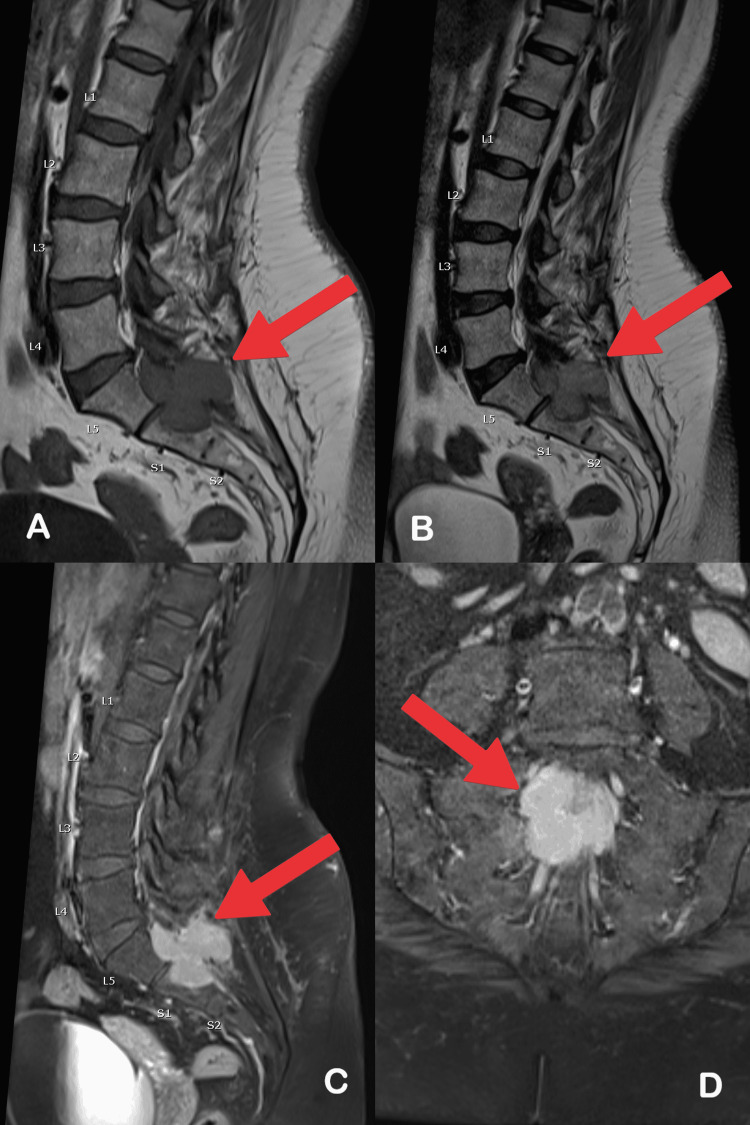
(A) Sagittal T2, (B) Sagittal T1, (C) Sagittal post-contrast, and (D) Axial post-contrast MRI images of the lumbosacral spine demonstrating large, lobulated, expansile, intradural, T2 hyperintense, T1 isointense, enhancing soft-tissue mass located at the level of L5-S1. Adjacent vertebrae are intact. There is severe bone remodelling at the posterior aspect of S1 and S2 vertebrae along with posterior soft-tissue invasion.

A 52-year-old female with left breast carcinoma presented with long-standing anal and sacral pain. The patient was alert and oriented. There were no cerebellar or meningeal signs. She had a completely normal physical/system examination. MRI Spine showed a multilobulated mass lesion located in the sacral spinal canal at the level of S1 and S2 causing extrinsic bony erosions in the posterior aspect of the body of S1 and S2 with the posterior extra spinal extension of the lesion through the posterior arch of the S1 and S2 vertebrae. The proximal part of the lesion appeared to be intradural in location and the distal part of the lesion appears intradural and extramedullary in location. The lesion showed intermediate signal intensity on T1 weighted images and bright signal intensity on short tau inversion recovery (STIR) sequences with enhancement on post-contrast images (Figure 1). Sacral lesion biopsy and histopathological evaluation revealed Rosai-Dorfman disease. Steroid therapy was started for the patient. The patient responded well on follow-up and stopped steroids after four months.

## Discussion

RDD or sinus histiocytosis with massive lymphadenopathy or Rosai-Dorfman-Destombes disease is a rare reactive histiocytic disease. RDD is specified by the overproduction of histiocytes in the lymph nodes [1]. It is distinguished by an array of symptoms, which include painless bilateral cervical lymph nodes enlargement, weight loss, raised temperatures, nasal obstruction, or tonsillar enlargement along with a multitude of other extranodal involvements [3,4]. Diagnosis is a combination of histopathological and radiological findings. 

Our case report is of a secluded spinal RDD. It can occur at any age but it most commonly involves patients between 20-40 years old and has a slight predominance in the male sex group. Viral, infectious, or autoimmune elements might be associated with causing it [3]. Lab findings are non-specific and may include raised ESR, neutrophilia, and polyclonal hyperglobulinemia. Spinal and central nervous system RDD are seen as dural-based lesions on imaging. RDD of the central nervous system, which did not include other extranodal sites, is observed only in 5% or fewer patients [5]. On T1-weighted imaging, the spinal or intracranial RDD can appear as isolated or numerous well-defined lesions that show iso to hyperintense signal intensity with post-contrast homogenous enhancement. On T2-weighted MRI RDD has a hypointense signal [6]. RDD is hard to detect only on imaging as the differentials such as lymphoma, metastasis, plasma cell granuloma, and meningioma have similar imaging features. RDD and meningiomas show similar postcontrast characteristics with homogenous enhancement. The differentiating feature, though, is the clear-cut dural tail sign of the meningioma. Metastasis shows hypointense T1 and hyperintense T2 weighted imaging. Lymphomas are mostly isointense on both T1 and T2 weighted imaging. Plasma cell granulomas show inhomogeneous enhancement with other similar imaging features.

Positron emission tomography (PET)/CT using 18F-FDG can also be used to diagnose RDD and show numerous enlargements, as described by Dhull et al. [7]. Histopathological findings of RDD entailing leptomeninges has characteristics close to the disease of the lymph nodes, thickened dura, permeation by lymphocytes, plasma cells, and pale histiocytes. The typical finding includes emperipolesis (lympho-phagocytosis) [8]. Mostly, spinal and intracranial RDD can be wrongly diagnosed and confused as a meningioma if histopathology is not done. Well-defined pale histiocytes are immunoreactive for S-100 protein and CD-68, and negative for EMA and CD1a are seen in RDD [9]. Treatment is not required in most cases. It is advised only in patients having symptoms or where the lesions are menacing other essential body organs. Surgery is the first and the most favoured mode of treatment for spinal and intracranial RDD, as it leads to diagnosis as well as quick relief of the symptoms [10,11]. In symptomatic patients steroids, radiotherapy, chemotherapy, supportive therapy, and α-interferons can be employed [12,13]. A wait-and-watch mode is commonly used after surgery [5].

## Conclusions

Isolated RDD chancing on its own is a very rare histiocytic disease along with it being an exceptionally difficult diagnosis. The diagnosis is mainly dependent on histological and immunophenotypical testing. Its radiologic features are similar to other dural-based lesions. Disease progression is extremely rare. Surgical treatment relieves the compressive symptoms and remains the treatment of choice. Neoadjuvant therapies, steroids, and other treatments are available, although further extensive systematic investigations should be made in this field.
